# *In Vitro* Isolation and Characterization of Oxazolidinone-Resistant Mycobacterium tuberculosis

**DOI:** 10.1128/AAC.01296-17

**Published:** 2017-09-22

**Authors:** Matthew B. McNeil, Devon D. Dennison, Catherine D. Shelton, Tanya Parish

**Affiliations:** TB Discovery Research, Infectious Disease Research Institute, Seattle, Washington, USA

**Keywords:** tuberculosis, oxazolidinones, linezolid, resistance, fitness, ribosome, Mycobacterium, Mycobacterium tuberculosis, antibiotic resistance, mycobacteria

## Abstract

Oxazolidinones are promising candidates for the treatment of Mycobacterium tuberculosis infections. We isolated linezolid-resistant strains from H37Rv (Euro-American) and HN878 (East-Asian) strains; resistance frequencies were similar in the two strains. Mutations were identified in ribosomal protein L3 (RplC) and the 23S rRNA (*rrl*). All mutant strains were cross resistant to sutezolid; a subset was cross resistant to chloramphenicol. Mutations in *rrl* led to growth impairment and decreased fitness that may limit spread in clinical settings.

## TEXT

The oxazolidinone class of antibiotics inhibits the formation of protein synthesis initiation complexes by binding to domain V of the 23S rRNA (1). Linezolid (LZD), the first member of the oxazolidinones approved for clinical use, has recently been investigated as a potential treatment for drug-resistant strains of Mycobacterium tuberculosis (2, 3). LZD demonstrates time-dependent kill kinetics against replicating M. tuberculosis (4), bactericidal activity against nonreplicating bacilli (5), and good efficacy in mouse models (5). However, the long-term administration of LZD is limited due to side effects that include neuropathy and anemia (2, 6). Sutezolid (SZD; previously PNU-100480) is a next-generation oxazolidinone that has improved tolerance over long-term administration and improved efficacy against M. tuberculosis in a mouse model (7). Resistance to oxazolidinones has been studied in other bacteria and is mediated via mutations in domain V of the 23S rRNA (*rrl*), in the ribosomal protein L3 (*rplC*), or by the transporter OptrA (8). We sought to further characterize the mechanisms of oxazolidinone resistance in M. tuberculosis.

We isolated resistant mutant strains (RMs) against LZD by plating late-log-phase cultures of M. tuberculosis H37Rv (ATCC 25618) (Euro-American lineage) and HN878 (East-Asian lineage) on Middlebrook 7H10 agar with 10% (vol/vol) OADC (oleic acid-albumin-dextrose-catalase) supplement (Becton Dickinson) and 8 μM (2.7 μg/ml) of LZD (5× MIC) (9). We confirmed resistance by determining MICs on solid medium or in liquid medium. Solid MICs were determined in 24-well plates on 7H10-OADC agar and were defined as the lowest concentrations that prevented growth. MIC_90_ was determined in Middlebrook 7H9 liquid medium with 10% (vol/vol) OADC and 0.05% (wt/vol) Tween 80 (Tw); bacterial growth was measured by the optical density at 590 nm (OD_590_) after 5 days, and the MIC_90_ was defined as the concentration at which 90% of growth was inhibited (10). Sixteen resistant strains were isolated in H37Rv at a frequency of 2.3 × 10^−9^ (Table 1); 12 resistant strains were isolated in HN878 at a similar frequency of 3.3 × 10^−9^ (Table 2). All strains were confirmed as resistant to LZD and were also cross resistant to sutezolid (SZD) (Tables 1 and 2).

**TABLE 1:** Oxazolidinone-resistant mutant strains of M. tuberculosis H37Rv

Strain	MIC on solid medium (μM)		L3 mutation	23S RNA mutation
	Linezolid	Sutezolid		
H37Rv	3.1	1.6	WT	WT
LP-LZD-RM2	50	12.5	WT	G2814T
LP-LZD-RM3	25	12.5	WT	G2814T
LP-LZD-RM4	50	12.5	WT	G2814T
LP-LZD-RM24	50	25	WT	G2814T
LP-LZD-RM1	50	12.5	C154R	WT
LP-LZD-RM11	50	12.5	C154R	WT
LP-LZD-RM12	50	12.5	C154R	WT
LP-LZD-RM13	50	12.5	C154R	WT
LP-LZD-RM14	50	12.5	C154R	WT
LP-LZD-RM15	50	12.5	C154R	WT
LP-LZD-RM16	50	12.5	C154R	WT
LP-LZD-RM17	50	12.5	C154R	WT
LP-LZD-RM21	50	12.5	C154R	WT
LP-LZD-RM22	50	12.5	C154R	WT
LP-LZD-RM23	50	12.5	C154R	WT
LP-LZD-RM25	50	12.5	C154R	WT
HN878 WT	1.6	0.4	WT	WT

**TABLE 2:** Oxazolidinone-resistant mutant strains of M. tuberculosis HN878

Strain	MIC_90_ in liquid medium (μM)^*a*^					L3 mutation	23S RNA mutation
	LZD	SZD	CM	GM	KM		
HN878 WT	3	2	7	3	1	WT	WT
HN-LZD-RM3	156	75	12	4	3	WT	G2299T
HN-LZD-RM5	65	81				WT	G2299T
HN-LZD-RM6						WT	G2299T
HN-LZD-RM9	107	61				WT	G2299T
HN-LZD-RM10						WT	G2299T
HN-LZD-RM13	92	62				WT	G2299T
HN-LZD-RM14						WT	G2299T
HN-LZD-RM1	60	31	412	3	2	WT	A2689T
HN-LZD-RM11	94	62	112	3	4	WT	G2814T
HN-LZD-RM2						WT	G2814T
HN-LZD-RM4						WT	G2814T
HN-LZD-RM8						WT	G2814T
H37Rv WT	3.1	1.6				WT	WT

a LZD, linezolid; SZD, sutezolid; CM, chloramphenicol; GM, gentamicin; KM, kanamycin.

We sequenced *rrl* and ribosomal protein L3 using primers TB-rrl-MMF1, CACACTGTTGGGTCCTGA; TB-rrl-MMF2, TGGAATCCGCTGTGAA; TB-rrl-MMF3, CAGGAGGTTGGCTTAGAA; TB-rrl-MMF4, TCGTGAACACCCTTGC; and TB-rrl-MMR1, CGCCGTAACTCTATGCA for *rrl* and primers TB-rplC-MMF1, TCGAGATGCGCACAC; and TB-rplC-MMR1, GGACGTCGAACAGCTC for *rplC*. In H37Rv, 12 strains had a C154R mutation in ribosomal protein L3 (L3_C154R_); four RMs had a G2814T mutation in *rrl*, equivalent to G2576T in Escherichia coli (Table 1). Of note, L3_C154R_ is the dominant mutation observed in LZD-resistant clinical isolates (6, 11–13). In contrast, all of our strain HN878 resistant isolates selected *in vitro* had mutations in *rrl*; these were G2299T, A2689T, or G2814T, equivalent to G2061T, A2451T, and G2576T in E. coli, respectively (Table 2). Mutations A2689 and G2299 are located in the LZD binding site of the 23S rRNA, with A2689 being a conserved residue that is functionally important for the peptidyl-transferase activity of the ribosome (14–16). G2814 stacks on top of the active site nucleotide G2743 (equivalent to G2505 in E. coli); therefore, mutations in G2814 are likely to disrupt the LZD binding site (14). G2814T and G2299T were previously observed in M. tuberculosis LZD^r^ strains isolated *in vitro* and *in vivo* (6, 17, 18). To the best of our knowledge, this is the first description of the A-to-T change at position 2689 encoded by *rrl* (*rrl*_A2689T_) being associated with LZD resistance.

**FIG 1:**
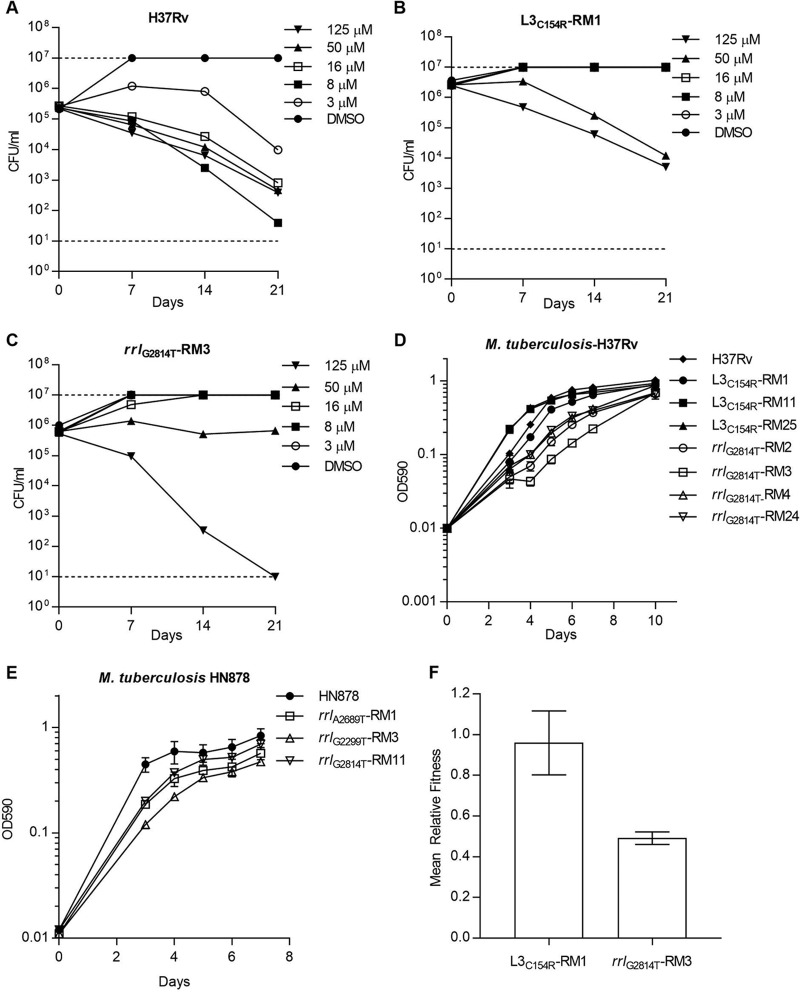
Phenotypes of LZD-resistant M. tuberculosis. *In vitro* kill kinetics of LZD against the WT (A), L3_C154R_ (B), and *rrl*_G2814T_ (C) strains of M. tuberculosis H37Rv. Growth of mutant strains in liquid medium in an M. tuberculosis H37Rv background (D) and an M. tuberculosis HN878 background (E). Results are the means and standard deviations from three biological replicates. (F) Fitness of the H37Rv L3_C154R_ and *rrl*_G2814T_ strains relative to that of the parental WT strain as determined by *in vitro* coculture experiments. Results are the means and standard deviations from three biological replicates. DMSO, dimethyl sulfoxide.

We determined bactericidal activity for LZD against wild-type (WT) and resistant strains. Bacterial viability was monitored over 21 d under replicating conditions using exponential-phase cultures of M. tuberculosis (5 × 10^5^ CFU/ml) in 7H9-OADC-Tw. CFU were counted by serial dilution and plating after 3 to 4 weeks of incubation at 37°C. LZD demonstrated bactericidal activity against WT H37Rv with a minimum bactericidal concentration (MBC) equivalent to the MIC (3 μM or 1 μg/ml) (Fig. 1A). The L3_C154R_ and *rrl*_G2814T_ mutant strains were resistant to LZD bactericidal activity up to 16 μM (5.4 μg/ml) (Fig. 1B and C). At 50 μM (17 μg/ml), LZD had activity against the L3_C154R_ strains and was bacteriostatic against the *rrl*_G2814T_ strain. Full kill was achieved against all strains at 125 μM (42 μg/ml). Thus, the increased MICs of L3 and *rrl* mutant strains were translated into proportional increases in MBCs.

Since LZD and chloramphenicol share a binding site in the 23S rRNA (14), we looked at cross-resistance. Strains carrying *rrl*_A2689T_ or *rrl*_G2814T_ were cross resistant to chloramphenicol, while *rrl*_G2299T_ did not confer resistance (Table 2). As expected, *rrl* mutant strains remained susceptible to kanamycin and gentamicin, both of which bind to the 16S rRNA (Table 2). Cross-resistance to chloramphenicol is consistent with data from Staphylococcus aureus (15, 19) and Mycobacterium smegmatis (20). However, the lack of cross-resistance for *rrl*_G2299T_ strains is surprising since the equivalent nucleotide in E. coli (G2061) interacts with the hydroxyl group of chloramphenicol (21). Furthermore, mutations of G2061 and A2062 are associated with chloramphenicol resistance in Thermus thermophilus (22). These results suggest that there are sufficient structural differences in the 23S rRNA of M. tuberculosis that cause chloramphenicol to bind in a different manner.

Mutations in 23S rRNA are commonly associated with growth defects in a diverse range of bacterial species, including M. smegmatis and M. tuberculosis (17, 19, 23). We conducted growth curves for representative strains in liquid medium; cultures were grown in 16-mm borosilicate tubes containing 5 ml of 7H9-OADC-Tw and incubated at 37°C with stirring at 250 rpm using an 8-mm stirrer bar. All strains with *rrl* mutations demonstrated impaired growth compared to WT strains (Fig. 1D and E), while strains with mutations in L3 were unimpaired (Fig. 1D).

The fitness cost of resistance mutations is an important contributor to the emergence and expansion of drug-resistant strains (24, 25). To investigate the fitness cost of LZD resistance, we conducted *in vitro* competition experiments as described previously (25). Briefly, 100 ml of 7H9-OADC was inoculated with ∼10^6^ CFU of the WT and mutant strains in a 450-cm^2^ roller bottle. Coculture experiments were grown at 37° until stationary phase (OD_590_, ∼1). Serial dilutions were plated onto 7H10-OADC with and without 5 μM (1.7 μg/ml) LZD at day 0 and at stationary phase. CFU were counted after 4 to 5 weeks of incubation at 37°C. The relative fitness (W) of resistant (R) compared to that of susceptible (S) strains was calculated by W = ln(R^F^/R^I^)/ln(S^F^/S^I^) (25), where R^I^ and S^I^ are the number of resistant and susceptible cells at day 0, and R^F^ and S^F^ are the number of resistant and susceptible cells at stationary phase. Experiments were performed in biological triplicate. The H37Rv *rrl*_G2814T_ strain had a fitness cost compared to that of the susceptible parental strain (Fig. 1F). In contrast, the L3_C154R_ mutant strain had no fitness cost relative to that of the parent (Fig. 1F). Relative fitness costs have been previously shown to influence the spread of resistance, with low-cost resistance phenotypes being the most prevalent within clinical populations (25). From a limited number of clinical studies, the L3_C154R_ single nucleotide polymorphism (SNP) is more prevalent than *rrl* SNPs within LZD-resistant strains (6, 11, 13). Whether or not this is because of the associated fitness cost requires further investigation. Resistance defects can be overcome by compensatory mechanisms as, for example, in S. aureus where changes in the copy number of 23S rRNA can achieve a balance between fitness and resistance (19). M. tuberculosis is unique in that it contains only a single copy of 23S rRNA, so it may not have access to the same compensatory mutations. Identifying compensatory mechanisms that overcome the fitness defects associated with *rrl* SNPs would provide further insights.

In conclusion, we demonstrate that mutations in the 23S rRNA (*rrl*) and the ribosomal protein L3 (RplC) are associated with resistance to the oxazolidinones LZD and SZD. Resistance led to decreased bactericidal activity from LZD. Mutations in *rrl*, but not L3, had a competitive fitness cost *in vitro*, suggesting that their appearance may be limited in clinical settings.
